# Digital and telehealth cognitive behavioral therapy for insomnia in comorbid mood/anxiety disorders

**DOI:** 10.1192/j.eurpsy.2026.12094

**Published:** 2026-07-31

**Authors:** B. Mihaela, D. Jelaga

**Affiliations:** Department of Mental Health, Medical Psychology and Psychotherapy, Nicolae Testemițanu State University of Medicine and Pharmacy, Chisinau, Moldova, Republic of

## Abstract

**Introduction:**

Insomnia frequently co-occurs with depressive and anxiety disorders, worsening symptom severity and functioning. Therapist-delivered CBT-I via videoconference (telehealth CBT-I) and digital CBT-I (dCBT-I; web/app) scaled widely, yet effect sizes, completion and safety in comorbid samples remain variably reported.

**Objectives:**

(1) Quantify effectiveness on insomnia; (2) estimate effects on depressive/anxiety symptoms; (3) assess adherence and safety in adults with mood/anxiety comorbidity.

**Methods:**

Targeted searches (2020–2025) in MEDLINE/PubMed, Embase, PsycINFO, Web of Science; adults ≥18 y; clinical/community. Designs: meta-analyses, RCTs, pragmatic/observational cohorts, open trials (quantified). Outcomes: ISI response ≥7, remission (ISI<8), PHQ-9, GAD-7, completion/attrition, AEs. Comparators: TAU, wait-list, sleep-education, in-person CBT-I. De-duplicated 41; screened 41; full-text 21; included 12; excluded 9 (unclear comorbidity 4; non-quantifiable 3; paediatric/protocol 2). Population characteristics inconsistent; one large cohort mostly male veterans.

**Results:**

Telehealth CBT-I cohort: 66% response/43% remission; 56% completed ≥5/6; ΔISI −9.9; similar all-video vs mixed. Two RCTs: non-inferior to in-person (−7.5 vs −7.8, 3 mo; margin 3 ISI).

dCBT-I: 2023 meta-analysis—large insomnia effects (moderate-large) and moderate reductions in depression (~0.4 SD) and anxiety (~0.3 SD) to 6 mo vs TAU/wait-list/education. Smartphone RCT: sleep efficiency +10.7 pp (3 mo) and +9.4 pp (6 mo) vs education (transdiagnostic; comorbidity not consistently reported). Head-to-head (secondary care): fully automated dCBT-I inferior to individual CBT-I at 9 wk (response 43% vs 70%; remission 18% vs 52%) and ~33 wk (remission 24% vs 56%).

MDD+insomnia: CBT-I increased antidepressant response to 32% vs 17% (OR≈2.3; NNT≈8) and improved insomnia remission vs TAU/wait-list. GAD+insomnia (open): response 61%; remission 26–48%; AEs ~33%, typically mild/transient during sleep-restriction. Serious AEs unreported; AE reporting sparse/heterogeneous.

**Conclusions:**

Telehealth CBT-I non-inferior to in-person; achieves 66% response/43% remission in real-world care. dCBT-I improves insomnia and reduces depression (~0.4 SD) and anxiety (~0.3 SD); fully automated may underperform individual CBT-I, supporting guided/stepped-care. Implication: first-line in stepped-care; intensify for non-responders. Limitation: platform/comparator/subgroup heterogeneity and under-representation of women in veteran samples limit %-to-% comparisons/generalizability.

**Disclosure of Interest:**

None Declared

